# Cryptocaryone Promotes ROS-Dependent Antiproliferation and Apoptosis in Ovarian Cancer Cells

**DOI:** 10.3390/cells11040641

**Published:** 2022-02-12

**Authors:** Yu-Chieh Chen, Che-Wei Yang, Te-Fu Chan, Ammad Ahmad Farooqi, Hsun-Shuo Chang, Chia-Hung Yen, Ming-Yii Huang, Hsueh-Wei Chang

**Affiliations:** 1 Department of Obstetrics and Gynecology, Kaohsiung Medical University Hospital, Kaohsiung 80708, Taiwan; 890084@ms.kmuh.org.tw (Y.-C.C.); tefu.chan@msa.hinet.net (T.-F.C.); 2 Graduate Institute of Natural Products, Kaohsiung Medical University, Kaohsiung 80708, Taiwan; u110531013@gap.kmu.edu.tw (C.-W.Y.); hschang@kmu.edu.tw (H.-S.C.); chyen@kmu.edu.tw (C.-H.Y.); 3 Department of Obstetrics and Gynecology, Kaohsiung Medical University, Kaohsiung 80708, Taiwan; 4 Institute of Biomedical and Genetic Engineering (IBGE), Islamabad 54000, Pakistan; farooqiammadahmad@gmail.com; 5 School of Pharmacy, College of Pharmacy, Kaohsiung Medical University, Kaohsiung 80708, Taiwan; 6 Department of Radiation Oncology, Kaohsiung Medical University Hospital, Kaohsiung 80708, Taiwan; 7 Department of Radiation Oncology, Faculty of Medicine, College of Medicine, Kaohsiung Medical University, Kaohsiung 80708, Taiwan; 8 Center for Cancer Research, Kaohsiung Medical University, Kaohsiung 80708, Taiwan; 9 Department of Biomedical Science and Environmental Biology, College of Life Science, Kaohsiung Medical University, Kaohsiung 80708, Taiwan

**Keywords:** Cryptocaryone, ovarian cancer, apoptosis, DNA damage

## Abstract

Cryptocaryone (CPC) is a bioactive dihydrochalcone derived from *Cryptocarya* plants, and its antiproliferation was rarely reported, especially for ovarian cancer (OVCA). This study aimed to examine the regulation ability and mechanism of CPC on three histotypes of OVCA cells (SKOV3, TOV-21G, and TOV-112D). In a 24 h MTS assay, CPC showed antiproliferation effects to OVCA cells, i.e., IC_50_ values 1.5, 3, and 9.5 μM for TOV-21G, SKOV3, and TOV-112D cells. TOV-21G and SKOV3 cells showed hypersensitivity to CPC when applied for exposure time and concentration experiments. For biological processes, CPC stimulated the generation of reactive oxygen species and mitochondrial superoxide and promoted mitochondrial membrane potential dysfunction in TOV-21G and SKOV3 cells. Apoptosis was detected in OVCA cells through subG1 accumulation and annexin V staining. Apoptosis signaling such as caspase 3/7 activities, cleaved poly (ADP-ribose) polymerase, and caspase 3 expressions were upregulated by CPC. Specifically, the intrinsic and extrinsic apoptotic caspase 9 and caspase 8 were overexpressed in OVCA cells following CPC treatment. Moreover, CPC also stimulated DNA damages in terms of γH2AX expression and increased γH2AX foci. CPC also induced 8-hydroxy-2′-deoxyguanosine DNA damages. These CPC-associated principal biological processes were validated to be oxidative stress-dependent by *N*-acetylcysteine. In conclusion, CPC is a potential anti-OVCA natural product showing oxidative stress-dependent antiproliferation, apoptosis, and DNA damaging functions.

## 1. Introduction

Ovarian cancer (OVCA) commonly shows a poor prognosis when detected late. OVCA was listed as the five leading causes of cancer death in the United States by age and sex in 2018 [1]. In the United States 2021 Cancer Statistics [1], the estimated new cases were 21,410, and estimated deaths were 13,770 for OVCA patients. The heterogeneity of OVCA, derived from different histological types of its epithelial cells with varying profiles of mutation [2,3], challenges OVCA therapy. In addition to surgery, chemotherapy is a supportive treatment for OVCA therapy. However, chemotherapy is frequently associated with side effects [4] due to cytotoxic effects on normal tissues. Therefore, it is warranted to develop novel anticancer drugs that can effectively improve OVCA therapy.

Natural products may show antiproliferation of ovarian cancer cells [5,6] or reduce side effects of chemo-radiotherapy [7,8]. *Cryptocarya* plants are evergreen trees consisting of about 350 species, with mainly tropical and subtropical distribution. Extracts and bioactive compounds from *Cryptocarya* plants show anticancer effects in several cancer studies [9,10,11,12,13,14,15], with rare studies on OVCA cells.

Cryptocaryone (CPC), a natural dihydrochalcone, is one of the bioactive components in several species of *Cryptocarya* plants [9,16,17,18]. Its anticancer effects have been reported for leukemia [17] and colon [18] cancer cells. However, these studies have mainly reported CPC purification and cytotoxic effects, lacking information about the mechanisms of action.

Reactive oxygen species (ROS)-dependent antiproliferation is a potential strategy for cancer therapy [19,20,21]. Drugs generating ROS may provide cytotoxic effects to inhibit proliferation against cancer cells. Since CPC was reported to cause ROS generation [22], the antiproliferation effects of CPC to several cancer cells warrant a detailed validation. Currently, the anticancer mechanisms of CPC were reported in prostate [23] and oral [22] cancer cells involving apoptosis. However, the anticancer effects of CPC on OVCA cells remain unclear.

The present study aimed to evaluate the impact and mechanism of CPC on inhibiting cell proliferation of several OVCA cell lines (SKOV3, TOV-112D, and TOV-21G). Since OVCA is a heterogeneous tumor [24,25], several histotypes of OVCA lines such as SKOV3 for adenocarcinoma, TOV-112D for endometrioid carcinoma (ENOCa), and TOV-21G for clear cell carcinoma [26,27] were chosen for examining their responses to CPC.

## 2. Materials and Methods

### 2.1. CPC Isolation

The CPC has been isolated from the root of *C. concinna* by cold methanol extraction and chloroform/water partitioning, where the chloroform fraction was processed by silica gel column chromatography and elution by a chloroform-methanol gradient as described in our previous study [22]. NMR spectrum of CPC in Supplementary Figure S1) proved the purity of CPC is >95%.

### 2.2. Inhibitors for Oxidative Stress and Apoptosis

Pretreatments of the oxidative stress and apoptosis inhibitors such as *N*-acetylcysteine (NAC; 10 mM, 1 h) (Sigma-Aldrich, St. Louis, MO, USA) [28,29,30] and Z-VAD-FMK (ZVAD; 100 μM, 2 h) (Selleckchem; Houston, TX, USA) [31] were performed in OVCA cells before CPC posttreatment.

### 2.3. Cell Culture

Human OVCA line (SKOV3), purchased from ATCC (Manassas, VA, USA), was cultured in McCoy’s 5A medium (Sigma-Aldrich; St. Louis, MO, USA) containing 10% fetal bovine serum (FBS). Human Type I OVCA cell lines (TOV-21G and TOV-112D) [32], purchased from Bioresource Collection and Research Center (BCRC) (Hsinchu, Taiwan), were cultured in (MCDB105/Medium 199 1:1) medium (Gibco, Grand Island, NY, USA) containing 15% FBS. These cell cultures were supplemented with 100 U/mL penicillin and 100 μg/mL streptomycin.

### 2.4. Viability and Cell Cycle Assays

According to the user’s instruction, the cell viability was measured by Promega’s MTS kit (Madison, WI, USA) detecting by ELISA reader at 490 nm [33]. Cells were processed with fixation by 75% ethanol overnight and stained by 7-aminoactinmycin D (7AAD; final concentration 1 μg/mL, 30 min). The 7AAD-stained cellular DNA content was reflected in different cell cycle phases (Biotium, Inc., Hayward, CA, USA) [34], which was detected by an Accuri C6 flow cytometer (Becton-Dickinson, Mansfield, MA, USA) (FL1 channel).

### 2.5. Flow Cytometry and Immunoblotting for Apoptosis Assay

According to user instruction, a strong Biotech Annexin V/7AAD kit (Taipei, Taiwan) was utilized to monitor apoptosis [9] by incubating Annexin V-FITC (1:1000) and 7AAD (final concentration 1 μg/mL) for 30 min. The Accuri C6 flow cytometer detected both annexin V and 7AAD intensities (FL1 and FL3 channels). Annexin V-positive (+)/7ADD positive/negative (+/−) populations were calculated for apoptosis analysis.

For immunoblotting, apoptosis sampler antibody kit (Cell Signaling Technology, Inc., Danvers, MA, USA), including antibodies for cleaved poly (ADP-ribose) polymerase (c-PARP), cleaved caspases-3 (c-Cas 3), c-Cas 9, and c-Cas 8, was applied as described previously [35]. In brief, 36 μg protein was loaded in 10% SDS-PAGE gel, transferred to PVDF membrane, and blocked with 5% skim milk. Subsequently, primary antibodies 1:1000 and secondary antibody 1:10,000 were sequentially applied.

### 2.6. Reactive Oxygen Species (ROS) and Mitochondrial Superoxide (MitoSOX) Assays

After cell harvesting and washing, ROS and MitoSOX were probed by their reacting dyes, i.e., 2′,7′-dichlorodihydrofluorescein diacetate (DCFH-DA; final concentration 2 μM, 30 min) (Sigma-Aldrich; St. Louis, MO, USA) [36] and MitoSOX™ Red (final concentration 5 μM, 30 min) (Molecular Probes, Invitrogen, Eugene, OR, USA), respectively. After reactions, their chemical probes for ROS and MitoSOX became fluorophores, which the Accuri C6 flow cytometer was detecting (FL1 and FL3 channels).

### 2.7. Mitochondrial Membrane Potential (MMP) Assay

After cell harvesting and washing, MMP was probed by its indicator dye DiOC_2_ (3) (final concentration 50 nM, 20 min) (Invitrogen, San Diego, CA, USA) [22]. After reactions to mitochondria with active membrane potentials, its chemical probe became the specific red fluorophore, which the Accuri C6 flow cytometer detected (FL1 channel).

### 2.8. Real-Time PCR Detection of Antioxidant Genes

Total RNA extraction and reverse transcription were performed by Trizol reagent (Invitrogen) and OmniScript RT kit (Qiagen, Valencia, CA, USA) [37]. Real-time PCR was performed by touch-down program [38] to examine several antioxidant pathway genes [39], including superoxide dismutase 1 (*SOD1*), heme oxygenase 1 (*HMOX1*), and thioredoxin (*TXN*). Detailed primer sequences were mentioned previously [35]. A 2^−ΔΔCt^ method [40] was applied in gene expression calculation in reference to the *GAPDH* gene.

### 2.9. γH2AX Detection for DNA Damage Assay

After harvesting and washing, cells were processed by fixation with 75% ethanol overnight. The γH2AX, a phosphorylated form of H2AX, was recognized by p-Histone H2A.X primary antibody (Santa Cruz Biotechnology, Santa Cruz, CA, USA) (50× dilution, 1 h, 4 °C). Subsequently, the secondary antibody-modified with Alexa Fluor^®^488 (Cell Signaling Technology) (10,000× dilution, 30 min, RT) was used. Finally, cells were stained by 7AAD (final concentration 1 μg/mL, 30 min). Cells were resuspended in PBS. The Accuri C6 flow cytometer detected both γH2AX and 7AAD intensities (FL1 and FL3 channels). γH2AX (+)/7ADD (+) populations were calculated for γH2AX analysis.

The protocol for observing γH2AX foci was slightly modified [41]. In brief, cells were washed with PBS, fixed with 4% paraformaldehyde (Sigma-Aldrich) for 5 min, permeabilized with 0.5% Triton X-100/PBS for 5 min, and blocked by 1% bovine serum albumin (BSA) for 1 h. Subsequently, cells were incubated with the primary antibody for γH2AX (Santa Cruz, CA, USA) (1:400) for 1 h. After washing, cells were incubated with a secondary antibody with Alexa Fluor 488 label (1:500 dilution) and counterstained with Hoechst 33342 (Sigma-Aldrich) (1:1000 dilution) for 1 h before mounting. Images were captured by a DMi8 microscope (Leica Microsystems, Wetzlar, Germany).

### 2.10. 8-Hydroxy-2′-Deoxyguanosine (8-OHdG) Detection for DNA Damage Assay

After harvesting and washing, cells were processed by fixation with 75% ethanol overnight. The 8-OHdG, a common oxidized nucleoside of DNA, was recognized by 8-OHdG-FITC one-step antibody (100× dilution, 1 h, RT) (Santa Cruz Biotechnology, Santa Cruz, CA, USA). Cells were resuspended in PBS. The Accuri C6 flow cytometer detected 8-OHdG intensities. The 8-OHdG (+) populations were calculated for 8-OHdG analysis.

### 2.11. Statistical Analysis

Multi-comparisons using JMP12 software (SAS Institute, Cary, NC, USA) can examine the significance of the differences among different experiments, i.e., on one-way analysis of variance (ANOVA) using Tukey HSD post hoc test or Student’s *t*-test (western blot). Each experiment of bar graph was labeled with small case letters for multiple comparisons. Different treatments showing non-overlapping letters differ significantly (*p* < 0.05).

## 3. Results

### 3.1. CPC Inhibits Proliferation on OVCA Cells

**Figure 1:**
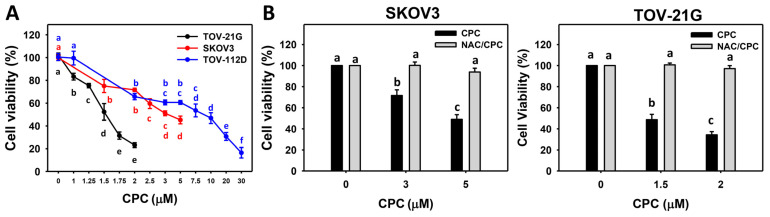
Cell viability of OVCA cells following CPC treatment. (**A**) 24 h MTS assay. Three OVCA cell lines (SKOV3, TOV-21G, and TOV-112D) were treated with vehicles (0 CPC containing 0.1% DMSO) and different concentrations of CPC for 24 h. (**B**) NAC recovered CPC-inhibited cell viability of OVCA cells. With NAC pretreatment or not, cells were exposed to 0, 3, and 5 μM (SKOV3) or 0, 1.5, and 2 μM (TOV-21G) of CPC for 24 h, namely NAC/CPC. Each experiment of bar graph was labeled with small case letters for multiple comparisons. Different treatments showed non-overlapping letters differ significantly (*p* < 0.05). For example (SKOV3 cells in (**B**)), the CPC (black column) 0, 3, and 5 show “a, b, and c” indicating significant differences between each other because they were non-overlapping with the same lower-case letter. In contrast, CPC 0 (black column) and NAC/CPC (gray columns) 0, 3, and 5 show “a” indicating nonsignificant differences among each other because they overlap with the same lower-case letter “a”. Data, means ± SD (*n* = 3).

Several histotypes of three OVCA cell lines (SKOV3, TOV-21G, and TOV-112D) were chosen for examining their antiproliferation effects on CPC. At 24 h exposure, the cell viabilities of these OVCA cells were decreased by CPC (Figure 1A). Among these cells, SKOV3 and TOV-21G cells exhibiting high sensitivities to CPC were used in the subsequent experiments to explore the antiproliferation mechanisms. Different concentrations of CPC with similar viabilities were chosen for examining their detailed mechanism, i.e., 0, 3, and 5 μM CPC (SKOV3) and 0, 1.5, and 2 μM CPC (TOV-21G).

Furthermore, the relationship between oxidative stress and antiproliferation effects on OVCA was evaluated by NAC. Following NAC pretreatment, CPC-induced antiproliferation effects on SKOV3 and TOV-21G cells were suppressed, revealing that oxidative stress may regulate antiproliferation by modulating related mechanisms. The possible mechanisms were examined in the following experiments.

### 3.2. CPC Modulates Cell Cycle Progressions on OVCA Cells

**Figure 2:**
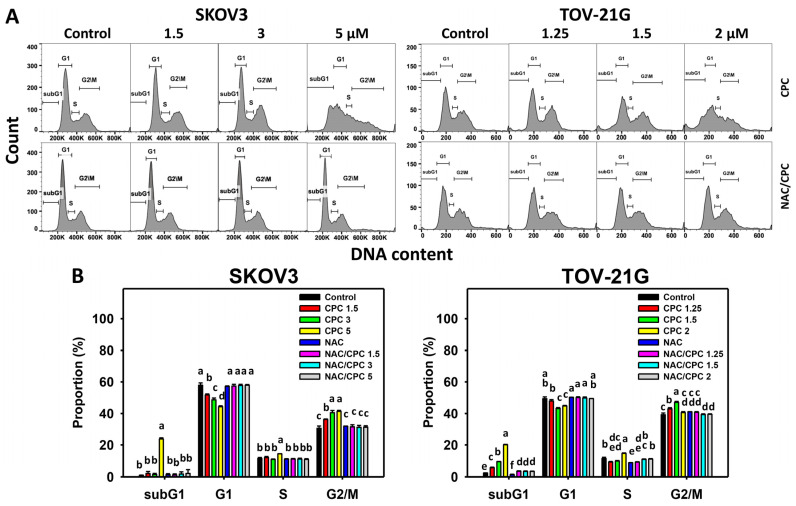
The cell cycle of OVCA cells following CPC treatment. (**A**) Cell cycle analysis and statistics. (**B**) NAC recovered CPC-induced subG1 accumulation of OVCA cells. With NAC pretreatment or not, cells were exposed to vehicle (0 CPC containing 0.1% DMSO), 3, and 5 μM (SKOV3) or 0, 1.5, and 2 μM (TOV-21G) of CPC for 24 h, namely NAC/CPC, i.e., NAC vs. control, NAC/CPC 1.5 vs. CPC 1.5, NAC/CPC 3 vs. CPC 3, and NAC/CPC 5 vs. CPC 5 (SKOV3) and NAC vs. control, NAC/CPC 1.25 vs. CPC 1.25, NAC/CPC 1.5 vs. CPC 1.5, and NAC/CPC 2 vs. CPC 2 (TOV-21G). Each experiment of bar graph was labeled with small case letters for multiple comparisons of the same cell phase. Different treatments showing non-overlapping letters differ significantly (*p* < 0.05). For example (TOV-21G cells in (**B**)), the CPC 0, 1.25, 1.5, and 2 show “e, c, b, and a” indicating significant differences between each other because they were non-overlapping with the same lower-case letter. Similarly, CPC at all concentrations (“e, c, b, and a”) was non-overlapping with the same lower-case letter of NAC/CPC at all concentrations (“f, d, d, and d”) indicating significant differences between CPC and NAC/CPC. In contrast, NAC/CPC 1.25, 1.5, and 2 show “d” indicating nonsignificant differences among each other because they overlap with the same lower-case letter “d”. Data, means ± SD (*n* = 3).

Cell cycle histograms of CPC at 24 h exposure to OVCA cells (SKOV3 and TOV-21G) were performed (Figure 2A). Following CPC treatment to OVCA cells, subG1 and G2/M populations were increased, while the G1 population was decreased as compared to the control (Figure 2B). These changes for cell cycle progression were alleviated by NAC pretreatment.

### 3.3. CPC Promotes Plasma Membrane-Detected Apoptosis on OVCA Cells

**Figure 3:**
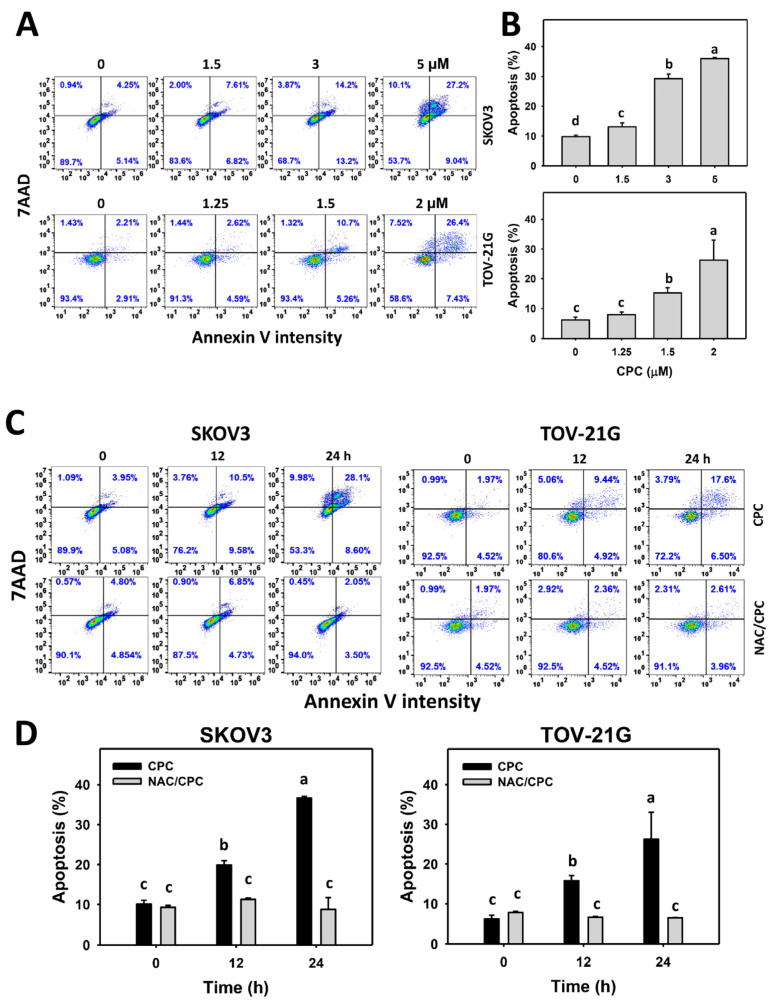
Annexin V measurement of OVCA cells following CPC treatment. (**A**,**B**) Annexin V/7AAD analysis and statistics. Cells were exposed to 0, 3, and 5 μM (SKOV3) or 0, 1.5, and 2 μM (TOV-21G) of CPC for 24 h. Annexin V (+)/7ADD (+/−) populations were calculated for apoptosis (+). (**C**) NAC suppressed CPC-induced annexin V of OVCA cells. With NAC pretreatment or not, cells were exposed to vehicle (0 CPC containing 0.1% DMSO) and 5 μM (SKOV3) or 0 and 2 μM (TOV-21G) of CPC for 0, 12, and 24 h, namely CPC and NAC/CPC. Each experiment of bar graph was labeled with small case letters for multiple comparisons. Different treatments showing non-overlapping letters differ significantly (*p* < 0.05). For example (SKOV3 cells in (**D**)), the CPC 0 h, CPC 12 h, and CPC 24 h show “c, b, and a” indicating significant differences because they were non-overlapping with the same lower-case letter. In contrast, CPC 0 h, NAC/CPC 0 h, NAC/CPC 12 h, and NAC/CPC 24 h show the same letter with “c” indicating nonsignificant differences between each other because they were overlapping with the same lower-case letter. Data, means ± SD (*n* = 3).

Annexin V/7AAD is an apoptosis detection assay based on the flip-flop character of plasma membrane phospholipid (phosphatidylserine) in apoptosis. Annexin V/7AAD histograms of CPC exposed to OVCA cells (SKOV3 and TOV-21G) were performed (Figure 3A,C). OVCA cells that were exposed to CPC at different concentrations demonstrated higher apoptosis (+) (annexin V (+)) (%) than control (Figure 3B). Similarly, OVCA cells that were exposed to fixed concentrations of CPC at different times demonstrated higher apoptosis (+) (annexin V (+)) (%) than the control (Figure 3D). These time-course changes for apoptosis to OVCA cells were alleviated by NAC pretreatment.

### 3.4. CPC Promotes Caspase Activation for Apoptosis on OVCA Cells

**Figure 4:**
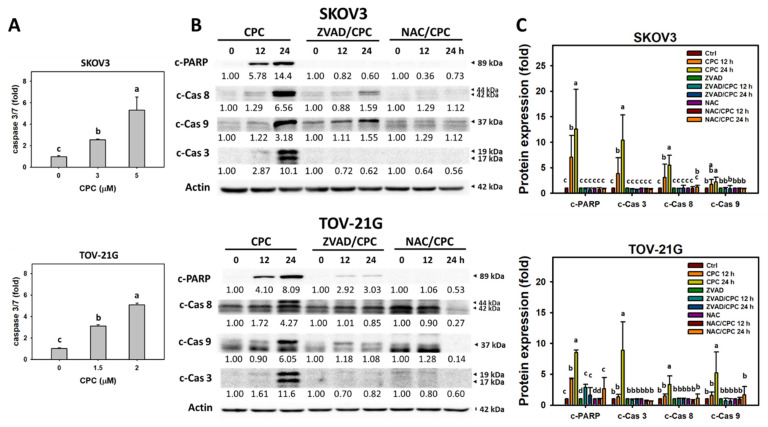
Apoptosis measurement of OVCA cells following CPC treatment. (**A**) Cas 3/7 analysis. Cells were exposed to 0, 3, and 5 μM (SKOV3) or 0, 1.5, and 2 μM (TOV-21G) of CPC for 24 h. (**B**,**C**) Immunoblotting for apoptosis proteins. With NAC pretreatment or not, cells were exposed to 5 μM (SKOV3) or 2 μM (TOV-21G) of CPC for 0, 12, and 24 h. Each experiment of bar graph was labeled with small case letters for multiple comparisons. Different treatments showed non-overlapping letters differ significantly (*p* < 0.05). Data, means ± SD (*n* = 3). For example (SKOV3 cells in (**C**)), the control, CPC 12 h, and CPC 24 h show “c, b, and a” indicating significant differences because they were non-overlapping with the same lower-case letter. In contrast, control, ZVAD, ZVAD/CPC 12 h, ZVAD/CPC 24 h, NAC, NAC/CPC 12 h, and NAC/CPC 24 h show the same letter with “c” indicating nonsignificant differences between each other because they were overlapping with the same lower-case letter.

Activation of several caspase networks was involved in triggering apoptosis [42]. Cas 3/7 activity was upregulated in OVCA following CPC treatment at different concentrations compared to the control (Figure 4A). In the immunoblotting assay, apoptosis-related proteins such as c-PARP and Cas 3/8/9 were upregulated in OVCA cells following CPC treatment at different times than the control (Figure 4B). These time-course changes for apoptosis protein expressions of OVCA cells were alleviated by NAC and ZVAD pretreatments, the inhibitors for oxidative stress and apoptosis.

### 3.5. CPC Promotes Oxidative Stress on OVCA Cells

**Figure 5:**
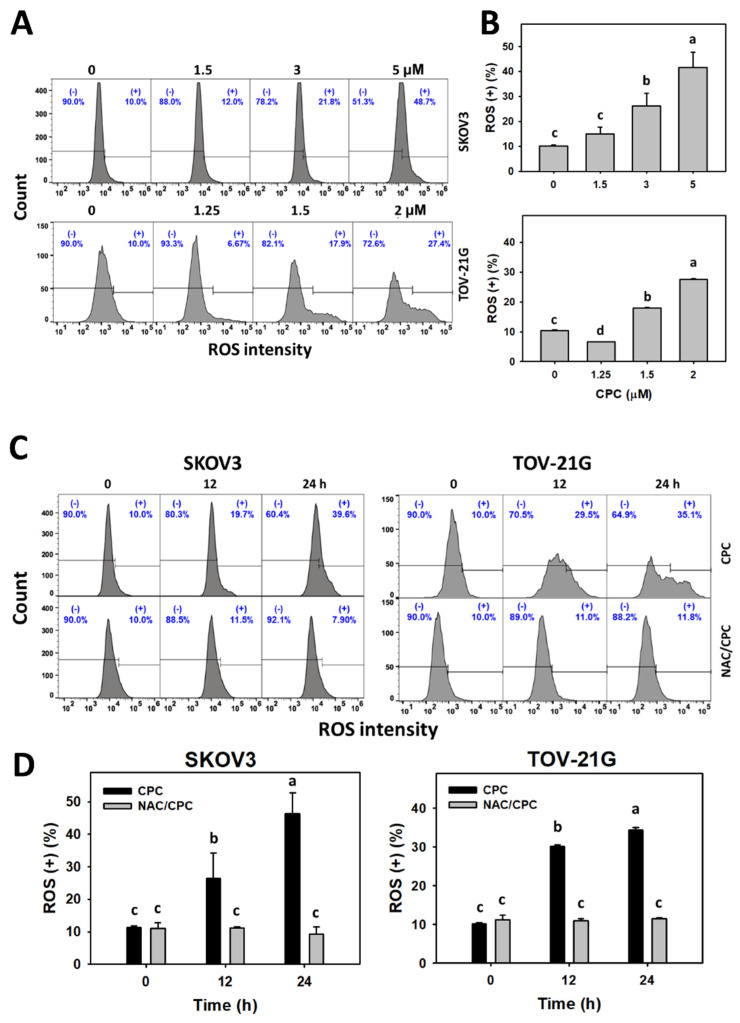
Oxidative stresses (ROS) measurement of OVCA cells following CPC treatment. (**A**,**B**) ROS analysis and statistics. Cells were exposed to 0, 3, and 5 μM (SKOV3) or 0, 1.5, and 2 μM (TOV-21G) of CPC for 24 h. Symbol (+) shown in each histogram indicates ROS (+). (**C**,**D**) NAC suppressed CPC-induced ROS of OVCA cells. With NAC pretreatment or not, cells were exposed to vehicle (0 CPC containing 0.1% DMSO) and 5 μM (SKOV3) or 0 and 2 μM (TOV-21G) of CPC for 0, 12, and 24 h, namely CPC and NAC/CPC. Each experiment of bar graph was labeled with small case letters for multiple comparisons. Different treatments showed non-overlapping letters differ significantly (*p* < 0.05). For example (TOV-21G cells in (**D**)), the CPC 0 h, CPC 12 h, and CPC 24 h show “c, b, and a” indicating significant differences because they were non-overlapping with the same lower-case letter. In contrast, CPC 0 h, NAC/CPC 0 h, NAC/CPC 12 h, and NAC/CPC 24 h show the same letter with “c” indicating nonsignificant differences between each other because they were overlapping with the same lower-case letter. Data, means ± SD (*n* = 3).

**Figure 6:**
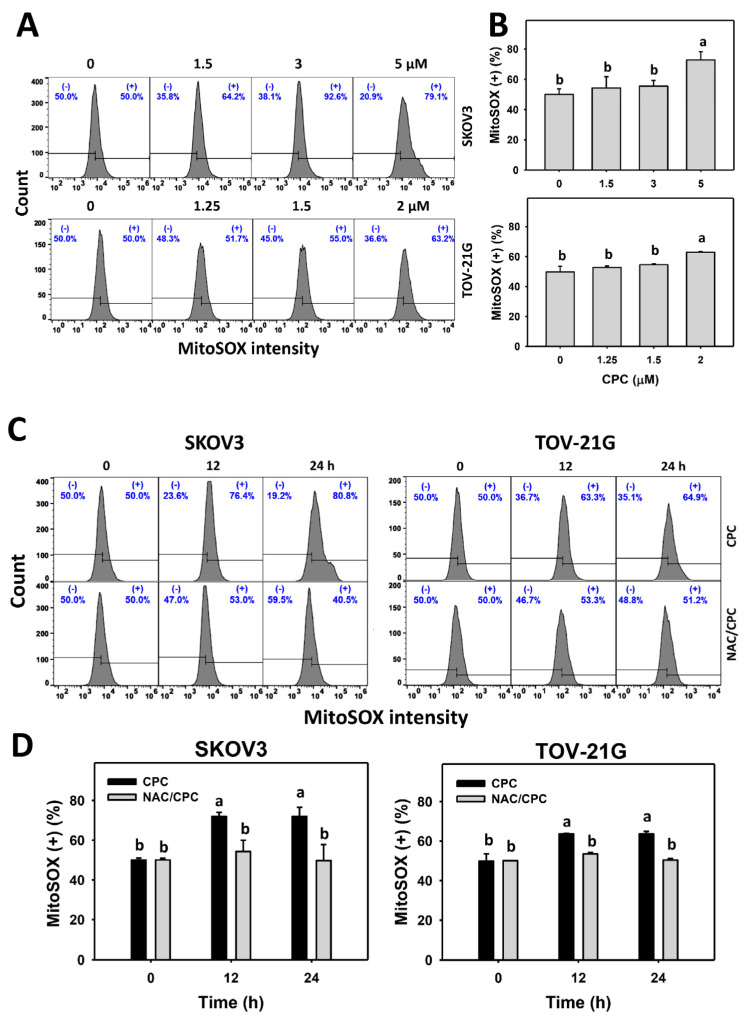
Oxidative stresses (MitoSOX) measurement of OVCA cells following CPC treatment. (**A**,**B**) MitoSOX analysis and statistics. Cells were exposed to 0, 3, and 5 μM (SKOV3) or 0, 1.5, and 2 μM (TOV-21G) of CPC for 24 h. Symbol (+) shown in each histogram indicates MitoSOX (+). (**C**,**D**) NAC suppressed CPC-induced MitoSOX of OVCA cells. With NAC pretreatment or not, cells were exposed to vehicle (0 CPC containing 0.1% DMSO) and 5 μM (SKOV3) or 0 and 2 μM (TOV-21G) of CPC for 0, 12, and 24 h, namely CPC and NAC/CPC. Each experiment of bar graph was labeled with small case letters for multiple comparisons. Different treatments showed non-overlapping letters differ significantly (*p* < 0.05). For example (SKOV3 cells in (**D**)), the CPC 0 h and CPC 12 h show “b and a” indicating significant differences because they were non-overlapping with the same lower-case letter. In contrast, CPC 0 h, NAC/CPC 0 h, NAC/CPC 12 h, and NAC/CPC 24 h show the same letter with “b” indicating nonsignificant differences between each other because they were overlapping with the same lower-case letter. Data, means ± SD (*n* = 3).

Although NAC reverted several changes as described above, the oxidative stress status of OVCA following CPC treatment still warrants detailed investigation. In the present study, ROS and MitoSOX statuses were observed to monitor oxidative stress changes. ROS and MitoSOX histograms of CPC exposed to OVCA cells (SKOV3 and TOV-21G) were performed (Figure 5A and Figure 6A). OVCA cells exposed to CPC at different concentrations demonstrated higher ROS and MitoSOX (+) (%) than control (Figure 5B and Figure 6B). Similarly, OVCA cells that were exposed to fixed concentrations of CPC at different times demonstrated higher ROS and MitoSOX (+) (%) than control (Figure 5C and Figure 6C). These time-course changes for oxidative stress to OVCA cells were alleviated by NAC pretreatment (Figure 5D and Figure 6D).

### 3.6. CPC Promotes MMP Dysfunction on OVCA Cells

**Figure 7:**
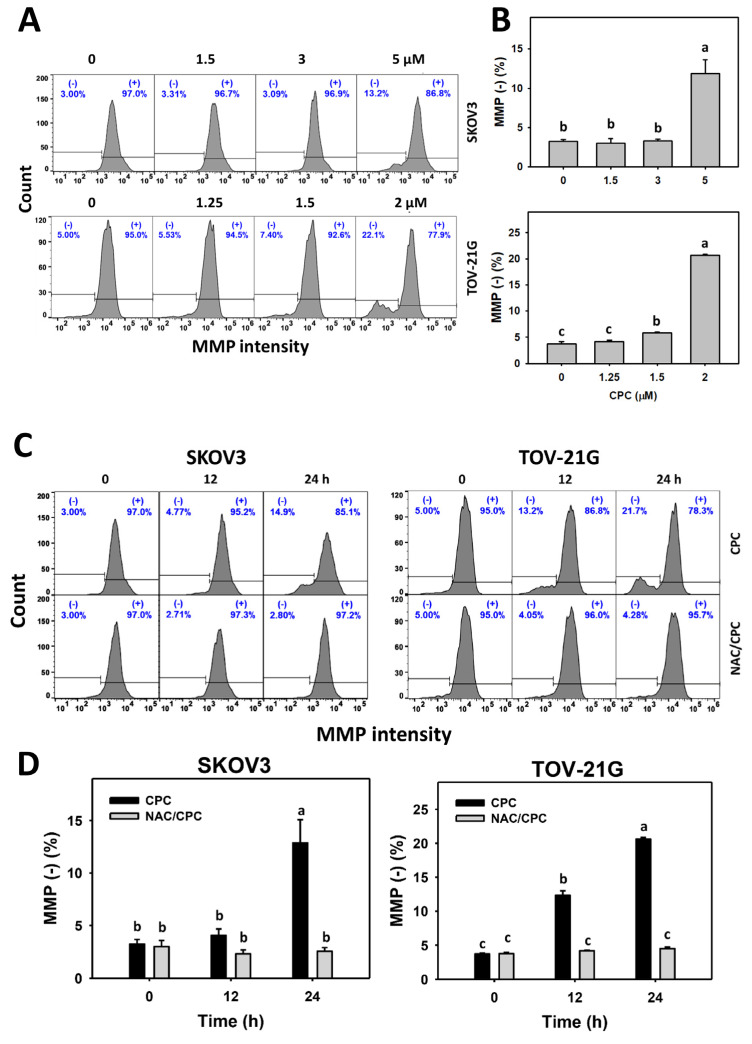
MMP measurement of OVCA cells following CPC treatment. (**A**,**B**) MMP analysis and statistics. Cells were exposed to 0, 3, and 5 μM (SKOV3) or 0, 1.5, and 2 μM (TOV-21G) of CPC for 24 h. Symbol (−shown in each histogram indicates MMP (−). (**C**,**D**) NAC suppressed CPC-induced MMP of OVCA cells. With NAC pretreatment or not, cells were exposed to vehicle (0 CPC containing 0.1% DMSO) and 5 μM (SKOV3) or 0 and 2 μM (TOV-21G) of CPC for 0, 12, and 24 h, i.e., namely CPC and NAC/CPC. Each bar of the bar charts was labeled with small case letters for multiple comparisons. Different treatments showing non-overlapping letters differ significantly (*p* < 0.05). For example (TOV-21G cells in (**D**)), the CPC 0 h, CPC 12 h, and CPC 24 h show “c, b, and a” indicating significant differences because they were non-overlapping with the same lower-case letter. In contrast, CPC 0 h, NAC/CPC 0 h, NAC/CPC 12 h, and NAC/CPC 24 h show the same letter with “c” indicating nonsignificant differences between each other because they were overlapping with the same lower-case letter. Data, means ± SD (*n* = 3).

In addition to ROS and MitoSOX, MMP is another reporter to monitor oxidative stress [43]. MMP histograms of CPC exposed to OVCA cells (SKOV3 and TOV-21G) were performed (Figure 7A,C). OVCA cells that were exposed to CPC at different concentrations demonstrated higher MMP (−) (%) than the control (Figure 7B). Similarly, OVCA cells that were exposed to fixed concentrations of CPC at different times demonstrated higher MMP (−) (%) than the control (Figure 7D). These time-course changes for MMP to OVCA cells were alleviated by NAC pretreatment.

### 3.7. CPC Promotes mRNA Expressions for Genes involved in Antioxidant Signaling on OVCA Cells

**Figure 8:**
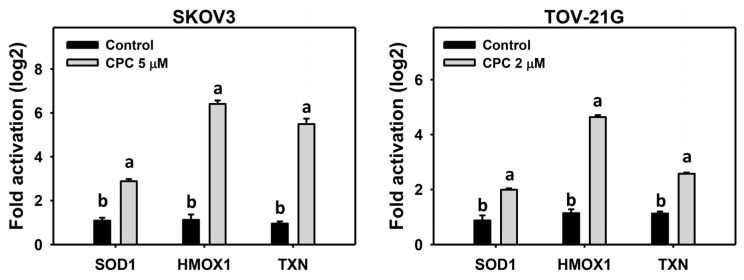
Antioxidant mRNA expression measurement of OVCA cells following CPC treatment. Cells were exposed to 0, 3, and 5 μM (SKOV3) or 0, 1.5, and 2 μM (TOV-21G) of CPC for 24 h. Antioxidant mRNA expressions were examined by real-time PCR. Each experiment of bar graph was labeled with small case letters for comparison of the same gene. Different treatments showing non-overlapping letters differ significantly (*p* < 0.05). For example (SKOV3 cells), control and CPC for all test genes show “b and a” indicating significant differences because they were non-overlapping with the same lower-case letter. Data, means ± SD (*n* = 3).

Oxidative stress may interact with cellular antioxidant signaling [44,45]. Several antioxidant genes, including *SOD1*, *TXN*, and *HMOX1*, were tested in OVCA cells following CPC treatment. These antioxidant mRNA expressions were generally promoted by CPC treatment in OVCA cells (Figure 8).

### 3.8. CPC Promotes DNA Damage on OVCA Cells

**Figure 9:**
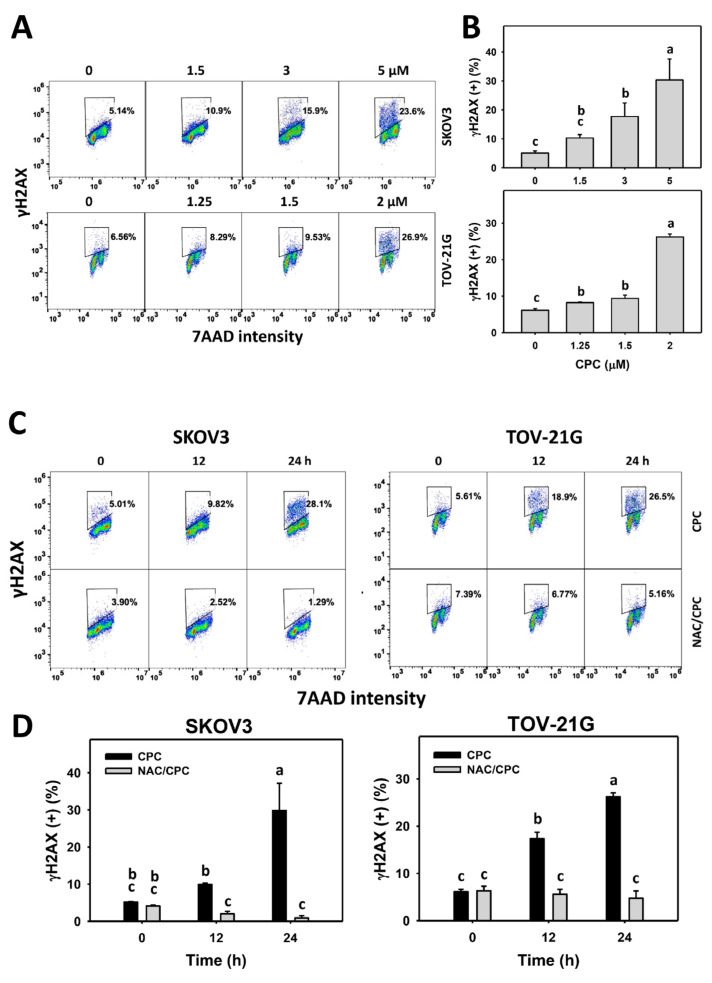

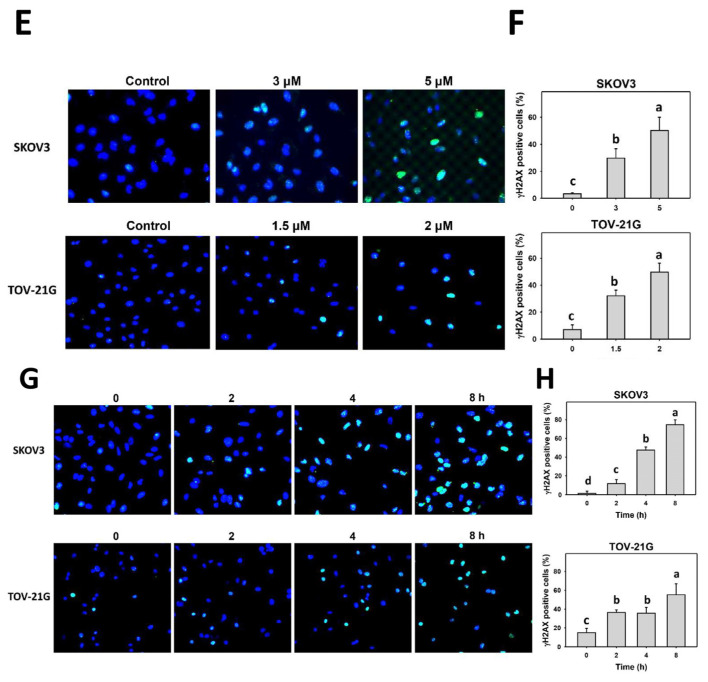
DNA damage (γH2AX) measurement of OVCA cells following CPC treatment. (**A**,**B**) γH2AX analysis and statistics. Cells were exposed to 0, 3, and 5 μM (SKOV3) or 0, 1.5, and 2 μM (TOV-21G) of CPC for 24 h. Symbol (+) shown in each histogram indicates γH2AX (+). (**C**,**D**) NAC suppressed CPC-induced γH2AX of OVCA cells. (**E**–**H**) Dose-response and time course for γH2AX foci. The γH2AX foci expressions were counted as γH2AX foci-positive cells (%). With NAC pretreatment or not, cells were exposed to vehicle (0 CPC containing 0.1% DMSO) and 5 μM (SKOV3) or 0 and 2 μM (TOV-21G) of CPC for 24 h, namely CPC and NAC/CPC. Each experiment of bar graph was labeled with small case letters for multiple comparisons. Different treatments showing non-overlapping letters differ significantly (*p* < 0.05). For example (SKOV3 cells in (**H**)), the CPC 0 h, CPC 2 h, CPC 4 h, and CPC 8 h show “d, c, b, and a” indicating significant differences because they were non-overlapping with the same lower-case letter. Data, means ± SD (*n* = 3 for (**A**–**D**) and *n* = 30 for (**E**–**H**).

**Figure 10:**
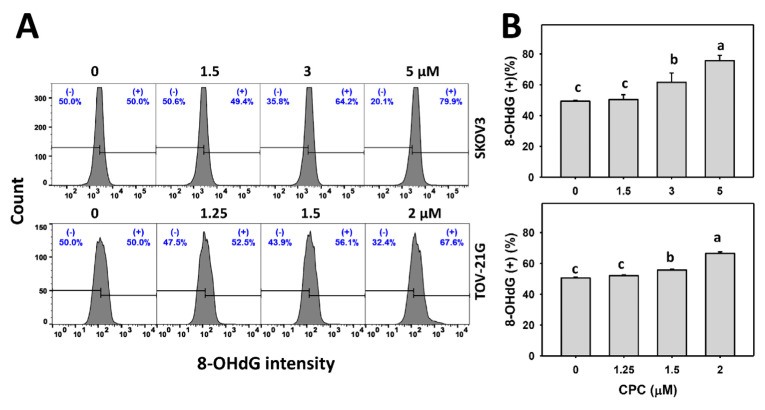

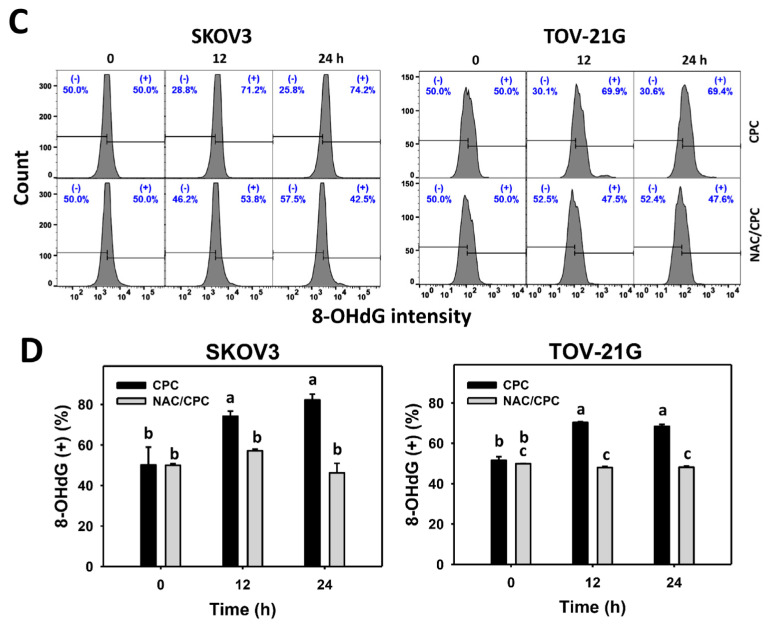
DNA damage (8-OHdG) measurement of OVCA cells following CPC treatment. (**A**,**B**) 8-OHdG analysis and statistics. Cells were exposed to 0, 3, and 5 μM (SKOV3) or 0, 1.5, and 2 μM (TOV-21G) of CPC for 24 h. Symbol (+) shown in each histogram indicates 8-OHdG (+). (**C**,**D**) NAC suppressed CPC-induced 8-OHdG of OVCA cells. With NAC pretreatment or not, cells were exposed to vehicle (0 CPC containing 0.1% DMSO) and 5 μM (SKOV3) or 0 and 2 μM (TOV-21G) of CPC for 0, 12, and 24 h, namely CPC and NAC/CPC. Each experiment of bar graph was labeled with small case letters for multiple comparisons. Different treatments showing non-overlapping letters differ significantly (*p* < 0.05). For example (SKOV3 cells in (**D**)), the CPC 0 h and CPC 12 h show “b and a” indicating significant differences because they were non-overlapping with the same lower-case letter. In contrast, CPC 0 h, NAC/CPC 0 h, NAC/CPC 12 h, and NAC/CPC 24 h show the same letter with “b” indicating nonsignificant differences between each other because they were overlapping with the same lower-case letter. Data, means ± SD (*n* = 3).

Both γH2AX and 8-OHdG statuses were observed for monitoring the DNA damage changes. γH2AX and 8-OHdG histograms of CPC exposed to OVCA cells (SKOV3 and TOV-21G) were performed (Figure 9A and Figure 10A). OVCA cells that were exposed to CPC at different concentrations demonstrated higher γH2AX and 8-OHdG (+) (%) than the control (Figure 9B and Figure 10B). Similarly, OVCA cells that were exposed to fixed concentrations of CPC at different times demonstrated higher γH2AX and 8-OHdG (+) (%) than the control (Figure 9C and Figure 10C). These time-course changes for DNA damage to OVCA cells were alleviated by NAC pretreatment (Figure 9D and Figure 10D).

Moreover, the number of γH2AX foci of CPC-treated OVCA cells was examined using immunofluorescence. γH2AX-based immunofluorescent patterns of OVCA cells following dose-response and time course of CPC treatments were demonstrated (Figure 9E,G). The γH2AX foci were dose- and time-dependently increased in OVCA cells (Figure 9F,H) following CPC treatments.

## 4. Discussion

Cryptocaryone (CPC) showed antiproliferation in some cancers (leukemia, prostate, colon, and oral) [17,18,22,23]. However, the anticancer effects of OVCA were rarely examined. Using PubMed searching, there were ten hits for “Cryptocaryone” (16 January 2022). Most of them were investigated for natural products from *Cryptocarya* species, which identified several bioactive compounds and reported the cytotoxicity for cancer cell lines without providing antiproliferation mechanisms. The present study, for the first time, reported the antiproliferation effect of CPC, being effective against three histotypes of OVCA cells, and examined the possible antiproliferation mechanisms.

### 4.1. CPC Exhibits Sensitive Antiproliferation Functions on OVCA Cells Attributed to Oxidative Stress

Three histotypes of OVCA cells were included in the present study, including SKOV3 for adenocarcinoma, TOV-112D for endometrioid carcinoma, and TOV-21G for clear cell carcinoma [26,27]. In the present study, the cytotoxicity values of CPC were 1.5, 3, and 9.5 μM for OVCA cells (TOV-21G, SKOV3, and TOV-112D) at 24 h MTS test (Figure 1). These results suggest that CPC effectively kills different types of OVCA cells.

Ovarian cancer cells (SKOV3, TOV-21G, and TOV-112D) exhibit different histotypes mentioned in Materials and Methods. The ATCC cell bank center reported that SKOV3 cells are resistant to clinical drugs such as cisplatin and adriamycin, whereas TOV-21G and TOV-112D are high-grade ovarian cancer cell lines. These ovarian cancer cells exhibited different chromosome abbreviations. These characteristics may contribute to different antiproliferation responses of CPC in different ovarian cancer cells. Moreover, various methods such as trypan blue staining exclusion can further confirm the antiproliferation difference between these cell lines in the future. In comparison, the cytotoxicity values of CPC for oral cancer cells (Ca9-22 and CAL 27) were 9.87 and 3.45 μM, respectively. These were less sensitive than OVCA cells.

Compared to clinical drugs for OVCA treatment, the first-line drug for OVCA, cisplatin [46], showed drug sensitivity of IC_50_ value with 3.17 μM at 72 h AlamarBlue assay [47]. Although different treatment times and viability assays were used, CPC effectively kills OVCA cells compared to cisplatin, i.e., 1.5 μM at 24 h MTS vs. 3.17 μM at 72 h AlamarBlue for SKOV3 cells. Moreover, SKOV3 cells exhibit drug resistance to anticancer drugs such as cisplatin and taxol [48]. Cisplatin was frequently reported with severe side effects [49], but CPC showed low cytotoxicity to normal oral cells (HGF-1) [22]. Since cytotoxic side effects on normal cells were low, CPC may provide an effective targeted drug candidate for the therapy to OVCA.

Since the ROS removing agent NAC can suppress the CPC-promoted antiproliferation of OVCA, ROS contributes to the antiproliferation regulation of OVCA.

### 4.2. CPC Stimulated Oxidative Stress-Related Changes on OVCA Cells

As mentioned above, the antiproliferation function of CPC to OVCA depends on oxidative stress. Consistently, oxidative stress such as ROS and MitoSOX were elevated by CPC in OVCA cells. Moreover, MMP dysfunction also contributes to oxidative stress [43]. NAC alleviated CPC-caused MMP dysfunction in OVCA cells. Therefore, CPC stimulates a comprehensive induction of oxidative stress on OVCA cells.

Moreover, different ovarian cell lines may show different ROS responses to the same drug treatments. For example, anti-malarial drug artesunate induced higher ROS generation in the ovarian cancer cells (HEY1 and HEY2) than SKOV3 cells [50]. Similarly, ROS induction for SKOV3 cells was higher than TOV-21G cells after CPC treatments (Figure 5). For comparison, CPC showed lower antiproliferation ability in SKOV3 cells than TOV-21G cells. Accordingly, the ROS increments cannot wholly reflect the antiproliferation for CPC treatment to ovarian cancer cells.

Moreover, oxidative stress is generally induced and accompanied by the simultaneous change of antioxidant response [51]. For example, cultured oocytes challenged with sustained oxidative stress upregulate *SOD1* mRNA expressions [52]. In UVC-irradiated mice, *SOD1* and *HMOX1* mRNA and protein expressions were induced [53]. Another antioxidant signaling, such as TXN [54], was upregulated by oxidative stress.

Similarly, CPC upregulated *SOD1*, *HMOX1*, and *TXN* mRNA expressions (Figure 7) in OVCA cells, accompanied by sustained oxidative stress (Figure 5 and Figure 6). These findings suggest that CPC induces antioxidant expressions but cannot offset the accumulated oxidative stress in OVCA cells.

### 4.3. CPC Stimulated Apoptosis on OVCA Cells Attributed to Oxidative Stress

Oxidative stress is essential to trigger apoptosis [55]. Similarly, CPC induces apoptotic changes such as subG1 accumulation and annexin V-detected flip-flop of phosphatidylserine in OVCA cells. These apoptosis outcomes were attributed to Cas 3/7 (Figure 4A). Since apoptosis may be turned on by two distinct pathways, such as intrinsic and extrinsic apoptosis, triggers, Cas 9 and Cas 8 activations were monitored in OVCA cells following CPC treatment. In addition to c-PARP and c-Cas 3 in immunoblotting, CPC upregulated the c-Cas 9 and c-Cas 8 expressions, the active forms for triggering apoptosis. Therefore, CPC triggered both intrinsic and extrinsic apoptosis in OVCA cells.

Furthermore, NAC can suppress the CPC-promoted annexin V- and western blot-detected apoptosis in OVCA cells (Figure 3 and Figure 4). NAC and ZVAD also downregulate the CPC-upregulated c-PARP, c-Cas 3, c-Cas 8, and c-Cas 9 expressions. Accordingly, ROS regulates intrinsic and extrinsic apoptosis in OVCA cells.

### 4.4. CPC Stimulated DNA Damage on OVCA Cells Attributed to Oxidative Stress

Oxidative stress is essential for initiating and promoting DNA damage [20], a common strategy for developing anticancer drugs [56]. Similarly, CPC stimulated oxidative stress-associated DNA damages by increasing γH2AX and 8-OHdG levels in OVCA cells (Figure 9 and Figure 10). To exclude the detection of non-targeted DNA damage by γH2AX, γH2AX foci were further monitored. Consistently, γH2AX foci were induced by CPC in OVCA cells. Furthermore, NAC can suppress CPC-promoted DNA damages in OVCA cells. Accordingly, ROS contributes to regulating DNA damage effects in OVCA cells.

## 5. Conclusions

The antiproliferation effect of a natural dihydrochalcone CPC was rarely investigated in OVCA cells. CPC was validated as a more effective antiproliferation agent to three types of OVCA cells than to oral cancer cells. Moreover, CPC is more effective in OVCA treatment than the first-line anticancer drug cisplatin. Mechanistically, CPC stimulated several kinds of oxidative stresses and promoted oxidative stress-associated apoptosis and DNA damages. This CPC-induced antiproliferation and oxidative stress responses were alleviated by NAC, demonstrating that CPC provided antiproliferation of OVCA cells in an oxidative stress-dependent way.

## Data Availability

Data are contained within the article.

## Supplementary Materials

The following are available online at https://www.mdpi.com/article/10.3390/cells11040641/s1, Figure S1: ^1^H NMR spectrum of CPC.
