# Supplementary figures and images for: Cryptocaryone Promotes ROS-Dependent Antiproliferation and Apoptosis in Ovarian Cancer Cells
Source: Cells. 2022 Feb 12;11(4):641. doi: 10.3390/cells11040641 (PMC8870566; doi:10.3390/cells11040641)

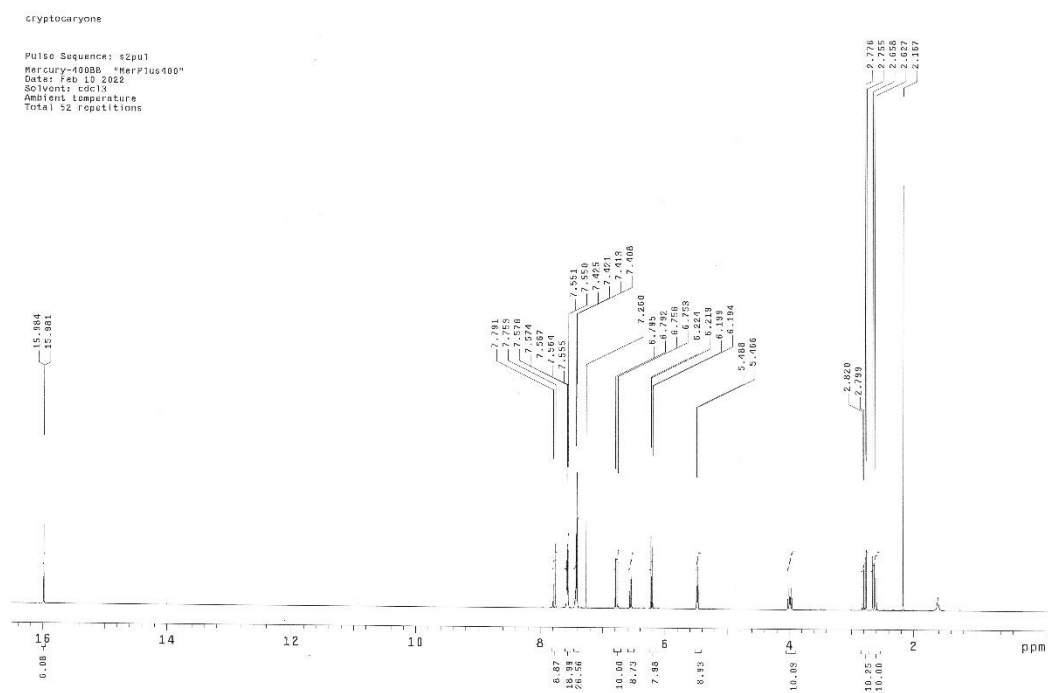

Supplementary Figure S1:  $^1\text{H}$  NMR spectrum of CPC.

Supplement: Supplementary file 1 [file cells-11-00641-s001.zip › cells-1585603-supplementary.pdf]
